# Cell Surface Charge Mapping Using a Microelectrode Array on ITO Substrate

**DOI:** 10.3390/cells12040518

**Published:** 2023-02-04

**Authors:** Leixin Ouyang, Rubia Shaik, Ruiting Xu, Ge Zhang, Jiang Zhe

**Affiliations:** 1 Department of Mechanical Engineering, University of Akron, Akron, OH 44325, USA; 2 Department of Biomedical Engineering, University of Akron, Akron, OH 44325, USA

**Keywords:** surface charge mapping, cell surface charge, photoelectric imaging, zeta potential

## Abstract

Many cellular functions are regulated by cell surface charges, such as intercellular signaling and metabolism. Noninvasive measurement of surface charge distribution of a single cell plays a vital role in understanding cellular functions via cell membranes. We report a method for cell surface charge mapping via photoelectric interactions. A cell is placed on an array of microelectrodes fabricated on a transparent ITO (indium tin oxide) surface. An incident light irradiates the ITO surface from the backside. Because of the influence of the cell surface charge (or zeta potential), the photocurrent and the absorption of the incident light are changed, inducing a magnitude change of the reflected light. Hence, the cell surface charge distribution can be quantified by analyzing the reflected light intensity. This method does not need physical or chemical modification of the cell surface. We validated this method using charged microparticles (MPs) and two types of cells, i.e., human dermal fibroblast cells (HDFs) and human mesenchymal stem cells (hMSC). The measured average zeta potentials were in good agreement with the standard electrophoresis light scattering method.

## 1. Introduction

Characterization of cell surface charge is essential for the understanding of numerous cellular behaviors [1,2,3]. Cell surface charge can affect many biological functions, such as cellular nutrition adsorption [4] and endocytosis [5,6]. Additionally, cell surface charge is highly asymmetric within a single cell and divergent among different cells [7]. Measurement of the cancer cell’s surface charge and its distribution can be used to understand fundamental metabolic mechanisms, clinical diagnostics, and therapeutics [8,9]. Nowadays, electrophoretic light scattering is a commonly used method for cell surface charge measurement [10]. However, this method measures the average bulk surface charge density or zeta potential of a group of cells, which cannot analyze individual cells. Ni et al. reported a microfluidic resistive pulse sensor that can evaluate individual cells’ surface charges and sizes [11]. However, it cannot map the surface charge distribution of a cell. Recently, several methods have been reported to probe and visualize surface charge distribution of cells [12], including atomic force microscopy (AFM) [13,14], scanning ion conductance microscopy (SICM) [15,16], and photoelectrochemical imaging systems (PEIS) [17,18,19]. AFM has been used to measure the cell surface charge by measuring the interaction force between the AFM tip and the local cell surface [20]. SICM positions a nanopipette close to the cell surface (e.g., within the electrical double layer) to measure the ionic current change caused by the local surface charge [16]. The PEIS employs a focused laser beam to scan a thin layer of n-type semiconductor material and generate a photocurrent. The local surface charge of a cell placed on the material can cause a photocurrent change [19,21]. Despite the surface charge mapping capability, all these mapping techniques rely on scanning the entire cell surfaces point by point via the precise control of a probe (e.g., an AFM tip, a nanopipette, and a focused laser beam) movement. Hence, mapping the surface charge of a cell with these methods tends to be too slow to perform rapid surface charge characterization. Recently, fluorescent nanoparticles (NPs) have been used as markers to investigate a single cell’s surface characterization [22,23]. Ouyang et al. reported a method of cell surface charge mapping via electrostatic cell-NP interactions [24]. The problem is that this method requires attaching charged nanoparticles to a cell, which may potentially change the cell’s surface property and affect the reuse of the cell for downstream applications.

Here, we report a method to map cell surface charge optically using a microelectrode array deposited on an ITO substrate. When a cell is positioned upon the microelectrode array, the local surface charge (or surface potential) affects the charge transfer from the solution to the microelectrodes and thus causes a change in the light absorption by the ITO substrate. Hence, by measuring the magnitude of the reflected light, the electrical potential change of each microelectrode (and thus the local zeta potential of the cell) can be determined. This method needs neither point-by-point cell surface scanning nor physical/chemical surface modification of the cell surface and can quickly map the surface charge of single cells.

## 2. Materials and Methods

### 2.1. Detection Principle and Measurement Setup

**Figure 1:**
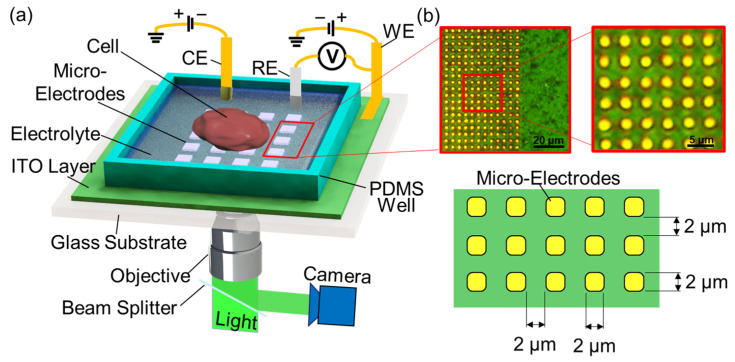
(**a**) Illustration of the method for rapid surface charge mapping. WE, RE, and CE are working, reference, and counter electrodes, respectively. The incident light illuminates the ITO layer from the backside. A cell sits on the surface of a microelectrode array. The local surface charge of the cell induces a change in the photoelectric effect. A CCD camera is used to capture the reflected light. (**b**) Microscopic images and scheme of the microelectrode array.

**Figure 2:**
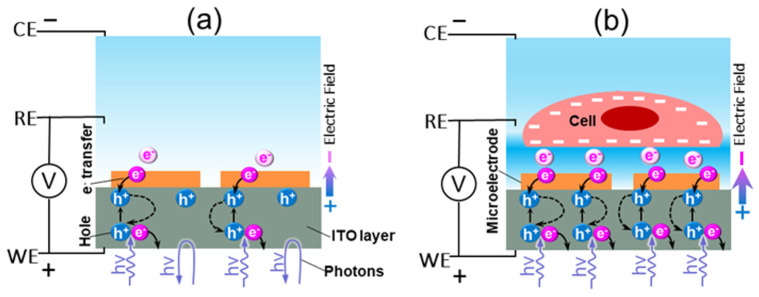
Illustration of cell surface charge mapping mechanism: (**a**) the photoelectric effect occurs in the microelectrode-ITO layer, which absorbs the incident light and reflects the unabsorbed photons; (**b**) the negatively charged cell surface enhances the local electric field, which increases the charge transfer and decreases the reflected light intensity.

As shown in Figure 1, the rapid measurement of single cell’s surface charge distribution is achieved by monitoring the intensity of the light reflected from the gold microelectrode array. When light is irradiated on an ITO-metal electrode, photons are absorbed, and electron-holes are generated. The absorbed photons are converted to electrical energy and photocurrents [25]. The voltage between the working electrode and the counter electrode generates an electric field between the ITO layer and the solution. Under this electrical field, excess electrons are transferred from the solution and diffuse toward the microelectrode array; at the same time, excess holes on the ITO layer, generated by the incident light, also migrate to the microelectrode array (see Figure 2A). The holes and the electrons combine near the microelectrode array. When a negatively charged cell (or a particle) is placed on the microelectrode array, it causes a decreased local potential in the solution near the microelectrode, which enhances the local electrical field as well as the charge transfer from the solution to the microelectrode/ITO layer, as shown in Figure 2B. Thus, excess holes in the ITO are reduced, and more photons are absorbed. As a result, the intensity of the reflected light is reduced. The lower the surface potential of the cell, the lower the intensity of the reflected light. Hence, the surface charge distribution of individual cells can be obtained by measuring the magnitude changes of the intensities of the reflected light beams from the microelectrode array.

The measurement setup is shown in Figure 1. A cell suspended in a buffer solution was placed on top of a microelectrode array consisting of 2 μm × 2 μm square microelectrodes made of gold. The microelectrode array was fabricated on an ITO substrate. A PDMS (polydimethylsiloxane) well was attached to the microelectrode-ITO layer to facilitate positioning cells on the microelectrode array. A light beam was emitted by a luminescent diode and focused on the microelectrode array from the backside by a microscope objective lens. A beam splitter was used to guide the reflected light to a CCD camera, where the reflected images were captured. A 0.1 Hz square wave voltage was applied between the working electrode and the solution. For each measurement, we recorded 2000 images continuously at a capturing rate of 20 Hz to capture the square-wave-shaped light intensity change of 10 cycles. The images taken from the CCD camera were then processed using a MATLAB program to generate a light-intensity map, which was subsequently converted to a surface charge (zeta potential) distribution. Gold electrodes were used to block the light transmitting through the electrode, which eliminated the error caused by the light absorption by the cell.

A three-electrode scheme was employed as shown in Figure 1 to vary the electrical potential applied to a solution. The ITO layer was used as the working electrode. A commercially available platinum foil (Fisher Scientific, Waltham, MA, USA) was used as the counter electrode. The reference electrode was a Ag/AgCl electrode (Fisher Scientific, Waltham, MA, USA). Both the counter and the reference electrodes were immersed into the buffer solution contained in the PDMS well. The reference electrode was placed approximately 1 mm above the microelectrode array. Square wave voltages were applied to the working electrode and the counter electrode to generate desired input signals for measurement and calibration. The voltage between the WE and the RE ($V=V_{w}-V_{re}$) was monitored by a data acquisition board (DAQ). The relationship between the reflected light intensity and the change in V was calibrated (see details in Section 2.4). After the calibration, cells suspended in buffer were loaded to the PDMS well. The surface charge measurement was then taken, after the cell settled on the microelectrode array. For the comparative purpose, cells from the same batch of culture were loaded to Zetasizer (Nano Z, Malvern Panalytical, Malvern, England, UK) to measure the average zeta potential. The concentrations of microparticles and cells in the solution were set to be ~10^5^/mL. The temperature was 25 °C, the refractive index for the buffer was 1.33, and 1.5 was used as an approximation for Henry’s function ƒ(κa).

### 2.2. Microelectrode Fabrication

A microelectrode array was fabricated on a 50.8 mm-by-50.8 mm ITO-glass slide (Fisher Scientific, Waltham, MA, USA). The thickness of the top ITO layer was 100 nm. First, the ITO-glass slide was washed in acetone and then rinsed with ultrapure water for 15 min. Next, a 20 nm layer of chromium and a 200 nm layer of gold were sputtered on top of the ITO layer in a Denton Desktop sputtering machine. Note that the 200 nm thick gold layer was used to prevent the transmission of incident light through the ITO layer. Next, the positive photoresist (AZ^®^ P4110) was spin-coated onto the gold surface, which was then soft baked for 5 min at 110 °C to consolidate the photoresist layer. The photoresist was exposed by an OAI mask aligner (Model 200) and was developed in an AZ^®^ 400K developer. A 20 min post-bake was applied to consolidate the photoresist layer. Subsequently, the gold and chromium layers were wet-etched in a gold etchant (44584; Alfa Aesar, Haverhill, MA, USA) and a chromium etchant (Type 1020AC; Transene Company Inc., Danvers, MA, US) separately to pattern the microelectrode array. Next, the ITO−glass slide was immersed into an AZ^®^ 400T Stripper to remove the remaining photoresist. As the last step, a PDMS well was fabricated and bonded to the ITO layer to hold the cells suspension upon the microelectrode array.

### 2.3. Cell Culture

Human dermal fibroblast cells (HDFs, Cat. No: CC-2511; Lonza, Basel, Switzerland) and human mesenchymal stem cells (hMSC, Cat. No: PT-2501; Lonza) were both cultured using Dulbecco’s modified eagle medium (DMEM, Cat. No: 11885084; ThermoFisher Scientific, Waltham, MA, USA) supplemented with 10% fetal bovine serum (FBS, Cat. No: A3160601; ThermoFisher) and 1% penicillin-streptomycin (Cat. No: 15140122; ThermoFisher). Briefly, 15 mL of complete medium was added to T75 flasks, and the cells were seeded at a density of 2500 cells/cm^2^ for HDFs and at a density of 5000 cells/cm^2^ for hMSC and incubated at 37 °C. After 16~24 h, the media were replaced. Then, they were changed every other day for HDFs and every 3 days for hMSC, until the flasks reached 90% confluency. After the flasks reached the desired confluency, the spent media were aspirated and the cells were washed once with 15 mL of Dulbecco’s phosphate-buffered salt solution 1X (DPBS 1X, Cat. No: 21031CV; Corning, Corning, NY, USA). Next, 4 mL of 0.25% Trypsin-EDTA solution (Cat. No: 25-053-CI, Corning) was added to HDFs and incubated at 37 °C for 5 min, whereas hMSC were harvested by adding 6 mL of Trypsin/EDTA for MSC (Cat. No: CC-3232; Lonza) and incubated at room temperature for no more than 15 min until the cells were detached from the surface. After trypsinization, complete DMEM medium was added to the flasks, and HDFs were pelleted using an Eppendorf centrifuge at 4 °C set at a speed of 200× *g* for 5 min, while hMSC were pelleted at room temperature at a speed of 600× *g* for 5 min. The cell pellets were counted by staining with Trypan blue solution (0.4%, Cat No: 17942E; Lonza) and resuspended in DPBS 1X solution to a final working concentration of 10^5^ cells/mL, which was used for the cell experiments.

### 2.4. Calibration of the Relation between the Potential and the Light Intensity

The reference electrode was placed approximately 1 mm above the microelectrode array. Hence, without a cell, we could use the potential at the reference electrode to represent the electric potential applied to the microelectrode array. A change in the electrical potential on the microelectrode surface would result in the intensity change of the reflected light. In the measurements, the light intensity reflected by the microelectrode array was significantly greater than that reflected by the blank ITO surface (i.e., the area without the microelectrodes). Hence, the reflection from the transparent ITO layer can be ignored.

**Figure 3:**
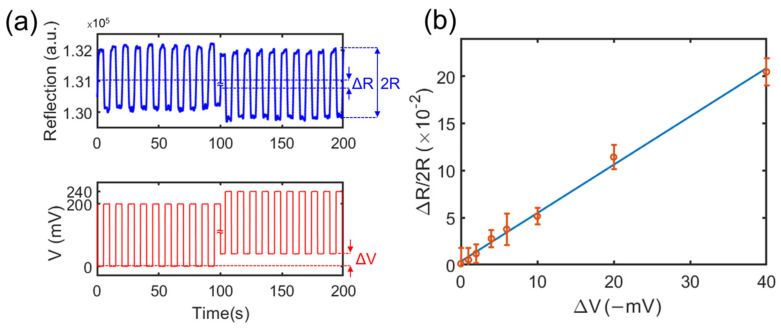
(**a**) Reflection intensity of a microelectrode with the changing of the applied voltage $V=V_{w}-V_{re}$, which is the voltage difference between the working and reference electrodes. The measurement was conducted in DI water. (**b**) The normalized reflection intensity changes as a function of the modulation amplitude Δ*V*.

We measured the reflected light intensity as a function of $V_{w}-V_{re}$ in DI (deionized) water. We applied a square wave signal across the working electrode and the counter electrode ($V_{w}-V_{c}$) with an amplitude of 200 mV, a frequency of 0.1 Hz of $V_{w}$, and a variable DC bias of $V_{c}$. Then, we measured the voltage between the working electrode and the reference electrode as $V=V_{w}-V_{re}$. At the same time, we monitored the reflected light intensity (Figure 3a). By varying the bias of $V_{c}$, V can be varied. ΔV was the difference between the ground potential (0 mV) and the minimum voltage of V, which represented the potential of the solution above the microelectrodes.

In the calibration, ΔV was varied from 0 to −40 mV, and the intensity change of the reflected light was measured to establish the relation curve. Figure 3a shows as a square wave V was applied, the reflected light intensity also exhibited a square wave shape. When ΔV was varied from 0 to −40 mV, we observed a decrease in the reflected light intensity. Here, we defined R as the difference between the highest reflection light intensity and the average reflection light intensity when a square waved V was applied, while 2R was the difference between the highest reflection intensity and the lowest reflection intensity. When a ΔV was changed, an average reflection intensity deference (ΔR) was observed. Figure 3b shows the relationship between the normalized reflected light intensity change (ΔR/2R) and ΔV. The error bars represent the standard variation of measured light intensity changes from 25 microelectrodes. The relation curve is linear. The correlation curve can be expressed as:

(1) ΔR/2R=K×ΔV

where K is a constant. Note that K is a function of the solution. From Figure 3b, the average value of K was determined to be 0.5 −V^−1^ for DI water. With the use of the microelectrode array, we can analyze the reflected light intensity from each 2 µm × 2 µm microelectrode. We also conducted the calibration for the cell culture medium (DPBS); the K value was determined to be 1.6 −V^−1^. In the next experiments with microparticles and cells, we used the calibration curves to convert the reflected light intensity from each microelectrode to the local potential of the measured object and obtain the mapping of the surface potential of the object.

## 3. Results

### 3.1. Validation of the Method via Charged Microparticles

**Figure 4:**
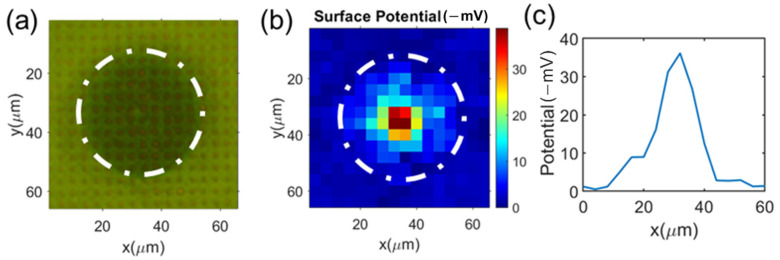
(**a**) Optical image (top illumination) of an MP sitting on the microelectrode array surface; (**b**) surface charge mapping measurement of this single MP using the microelectrode array; and (**c**) the profile of the measured surface electrical potential along the center line in the x-direction.

The microelectrode chip was placed under an inverted optical microscope. Polyethylene MPs with a size of 30 µm (BKPMS1.2; Cospheric LLC, Fisher Scientific, Waltham, MA, USA) were loaded to the PDMS well. The concentration of MPs was set to be approximately 10^5^/mL. After the MP settled on the bottom of the solution, a square wave voltage V (0 to 200 mV, 0.1 Hz) and ΔV = 0 mV was applied. The back illuminating light (through the ITO side to microelectrodes) was turned on. When an MP or a cell was positioned on the microelectrodes, it caused a local electrical potential change (local ΔV) upon each microelectrode, which eventually resulted in a decrease in the reflected light intensity. The reflected light/image was collected, and the intensity from each 2 µm by 2 µm electrode was analyzed by MATLAB. Note that the light intensity of the blank areas (areas between the microelectrodes) remained nearly unchanged. Figure 4a shows the corresponding optical image of an MP sitting on the microelectrode array with top illumination.

The electrical potential value on each 2 µm by 2 µm microelectrode (representing the local surface potentials of the cell surface) was obtained by converting the reflected light intensity using the calibration equation (Equation (1)). We assumed the surface potential obtained on each microelectrode represented a 4 µm by 4 µm square area centered around the 2 µm by 2 µm microelectrode. Figure 4b is the surface potential mapping measurement of the MP. The result shows that the highest surface potential occurred on the MP’s tip area. Away from the tip area, the potential gradually reduced. This can be explained as follows: since the MP was rigid, only its tip area was in contact with (or was very close to) the microelectrode array; hence, only in the tip area, the MP’s surface potential was close to the electrical potential of the microelectrodes. Away from the MP’s tip area, due to the increasing distance between the MP surface and the microelectrode, the influence of the MP’s surface potential on the microelectrode’s electrical potential was reduced [26].

**Figure 5:**
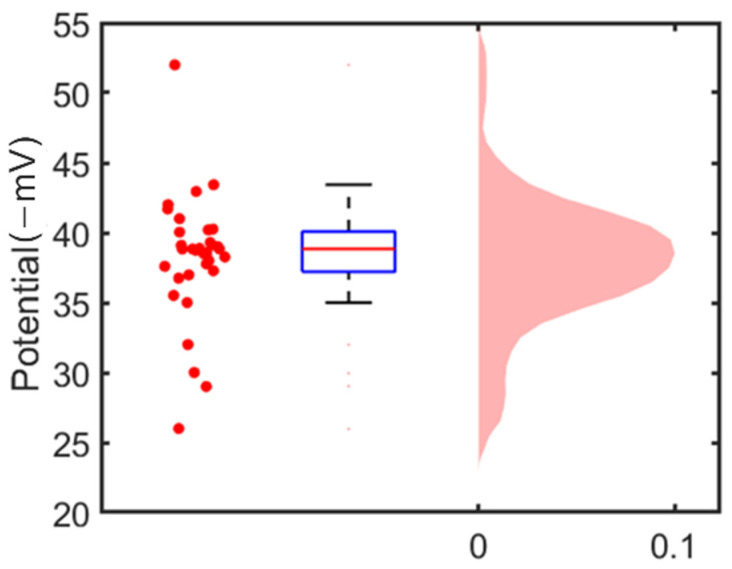
(Left) scattered plot of surface potentials of individual MPs; (center) boxplot of the surface potential measurement of MPs; (right) distribution of the surface potentials of the 30 MPs.

Figure 4c shows the surface potential along the centerline of the MP. The maximum electrical potential occurred at the MP’s tip area. The surface potential at the central electrode was −37 mV. We took additional measurements of 30 MPs sitting on the microelectrode surface. Because only at the tip area the measure electrical potential could represent the surface potential of MPs, for each mapping we only took the value from the central electrode (the highest value) to determine the MPs’ surface potentials. The average value of the 30 measured MP surface potentials was −38 ± 5 mV, as shown in Figure 5. To validate this measurement result, we measured the zeta potential of MPs from the same batch using the standard electrophoresis light scattering method (Nano Z, Malvern Panalytical, Malvern, England, UK); the result was −39 ± 5.41 mV. The two sets of results are consistent with each other. This test demonstrates the feasibility of mapping the surface charge of an MP or a cell with the microelectrode-ITO array.

Note that unlike rigid MPs, cells were soft, which could spread over and have good contact with the microelectrode array surface. Hence, we could use the microelectrode array chip to measure the surface charge mapping of cells.

### 3.2. Single Cell’s Surface Charge Mapping

Next, we measured the surface charge mapping of two different types of cells suspended in phosphate-buffered saline (DPBS) using the same microelectrode-ITO chip. HDFs and hMSC were chosen for the measurements. HDFs are common mammalian connective tissue cells with different shapes and functions under different conditions; this type of cell can repair and heal tissue, with potential applications in diagnostics and therapy [27]. hMSCs are used in cell-based therapy related to immune-mediated and degenerative diseases. They are capable of self-renewal and potential differentiation [28]. The surface charges of these cells can affect how the cells interact with their surroundings [29,30]; thus, measuring the surface potential distribution of these cells can benefit biomaterials research and cellular therapeutics. The K value for DPBS buffer, determined to be 1.6 −V^−1^, was used to back calculate the surface potential. A sample of the cell suspension containing the target cells was pipetted into the PDMS well. After ~5 min, we observed all cells settled on the microelectrode surface. Surface charge mapping via reflection measurement was then taken. Sixty HDFs cells and sixty hMSC cells were randomly selected for analysis. For each cell, we recorded 2000 images continuously at a capturing rate of 20 Hz to capture the light intensity change of 10 cycles. The images were processed to obtain the change in light intensity in the area where the cells were sitting.

**Figure 6:**
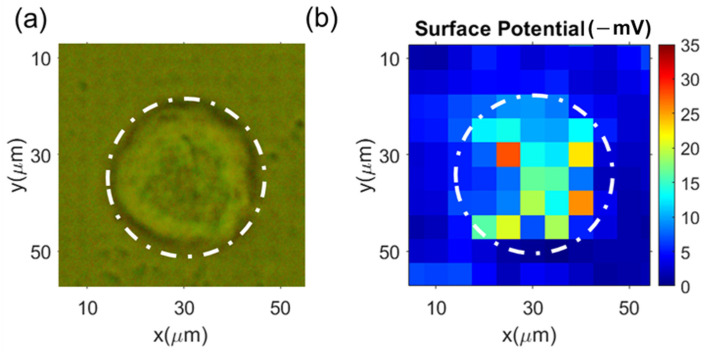
Typical measurement results of the surface charge mapping of HDFs: (**a**) optical images (top illumination) of the cells on the microelectrode array; (**b**) the corresponding surface charge mapping from the reflected images (measured from the back side of the microelectrode chip).

Typical results for HDFs are shown in Figure 6a, which shows the image of HDFs sitting on the microelectrode array with top illumination, while Figure 6b shows the surface potential mapping. From the mapping measurement, the average surface potential of these HDFs was −19 ± 7 mV. For the validation purpose, we also performed zeta potential measurements on the same batch of the HDFs using NanoZ, which gave the average zeta potential of −18.5 ± 2.4 mV. The mapping measurement via the microelectrode array matched well with the NanoZ measurement. From Figure 6b, we can identify the area that had a lower potential than the surrounding solution; this area matched with the actual cell area (Figure 6a) reasonably well.

**Figure 7:**
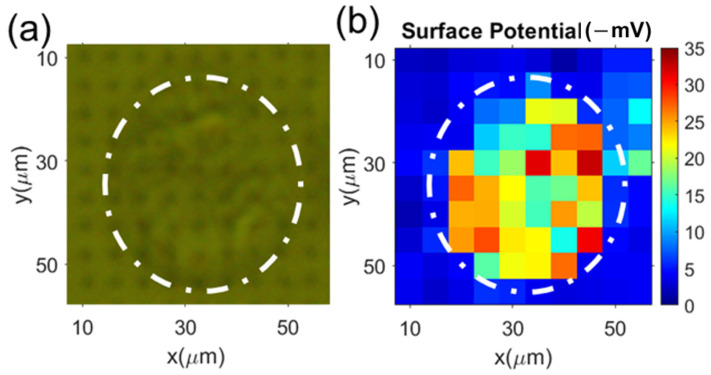
Typical measurement results of the surface charge mapping of hMSC: (**a**) optical images (top illumination) of the cells on the microelectrode array; (**b**) the corresponding surface charge mapping from the reflected images (measured from the back side of the microelectrode chip).

**Figure 8:**
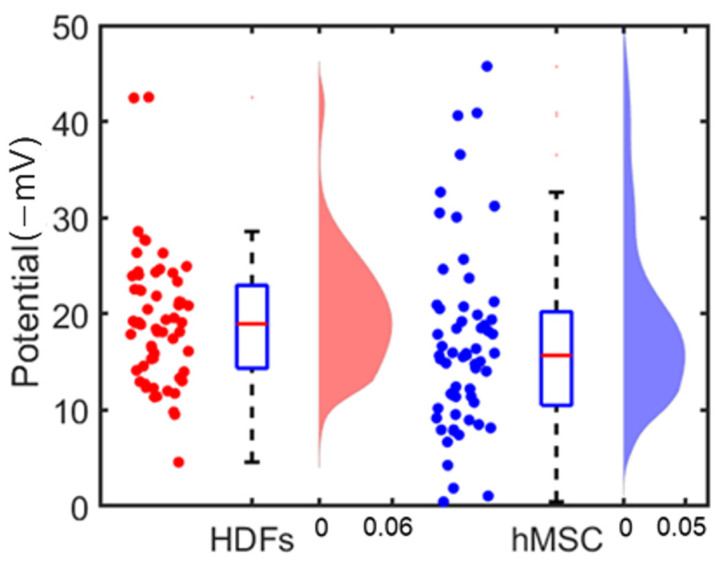
Comparison of the measured surface potentials of HDFs (red) and hMSC (blue). Left of each half-part: scattered plot of the surface potentials of individual cells; center of each half-part: boxplot of the surface potential measurement of cells; right of each half-part: distribution of the surface potentials of the 60 cells of each kind of cell.

Similarly, we conducted the surface charge mapping of the hMSC using the microelectrode array. The results are shown in Figure 7: Figure 7a shows the image of an hMSC cell on the microelectrode array; Figure 7b shows the cell’s surface potential mapping. The measured lower potential area also matched the actual cell location. Sixty hMSC were measured; the average surface potential of these cells was −17 ± 10 mV. This is in good agreement with the electrophoretic light scatting (NanoZ) measurement result, i.e., −15.6 ± 6.5 mV. The surface potentials of two types of cells measured from the surface charge mapping are plotted in Figure 8.

**Figure 9:**
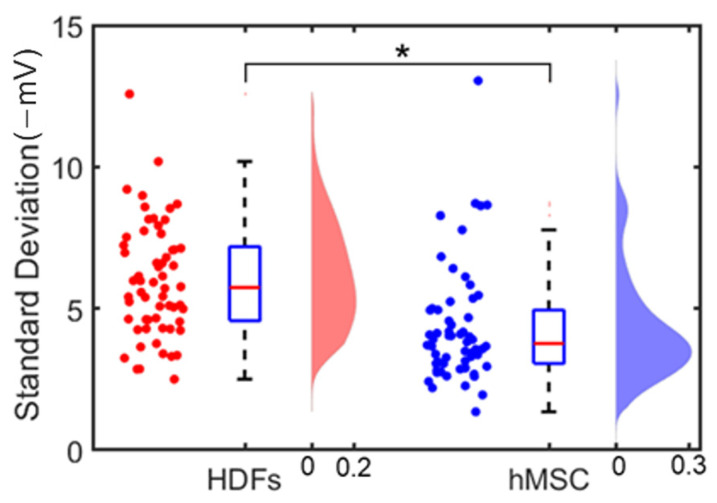
The standard deviations calculated from the surface potential distribution of single cells. Sixty independent cells of each cell type were analyzed. Two cell types were analyzed: HDFs (red) and hMSC (blue). (Left) the scattered plot of the standard deviations from single cells’ surface potential distribution; (center) the boxplot of the standard deviation from single cells’ surface potential distribution; (right) the histogram of the standard deviation from cells’ surface potential distribution. * represents *p* < 0.05 (for n = 60), which indicates a significant difference between the two groups of cells.

Figure 9 shows the standard deviations calculated from single cells’ surface potential distribution; each data point represents the standard deviation in surface potentials of all pixels within one cell. Sixty independent cells of each cell type were analyzed. The statistical analysis was carried out using MATLAB, and a *p*-value of less than 0.05 was set as a statistically significance level for the *t*-test. Figure 9 shows there was a distinct difference in the surface potential distribution patterns between the two types of cells: the averages of the standard deviations of HDFs’ and hMSC’ surface potential distributions were 5.9 and 4.3 (−mV), respectively. This indicates that HDFs had a less uniform surface potential distribution than hMSC had.

We demonstrated a new measurement method for cell surface charge mapping using a microelectrode array on an ITO surface. In this measurement, the cell surface charge distribution can be quickly determined by measuring the reflected light intensity without modifying the cell surface physically or chemically. Because of the small rigidity of cells, the cell surface charge mapping on the entire contact area can be enabled. However, for rigid objects, this method can only accurately measure the local potential in a close contact area [31]. It is worth noting that the irradiation intensity of the light source was less than 0.1 kW∙cm^−2^ and the illumination dose was ~20 kJ∙cm^−2^. This illumination dose is safe for keeping cells alive [32].

The spatial resolution of all measurements in this article was 4 µm × 4 µm because of the photolithography resolution in our lab. This resolution can be improved by patterning smaller electrodes (e.g., submicron squares using E-beam lithography), using higher-frequency laser with lower optical diffusion and higher-sensitivity photon detectors inducing less shot noise. The measurement precision was also estimated (i.e., the agreement between repeated measurements). We conducted surface charge measurements on one hMSC five times within 1000 s. The average zeta potential was −16 mV with a standard deviation of ±2.1 mV. Wu et al. used PEIS to measure the zeta potentials of individual hMSC four times; the standard deviation was ±2 mV [18]. Further, Zhu et al. used an optical absorption method based on an atomically thin molybdenum disulfide layer to measure the surface charge of protein binding. A standard deviation of ±1.2 mV was observed from multiple measurements [19]. The measurement precision of our method was similar to those of other methods. Note that a microelectrode array based on graphene and molybdenum disulfide were used for biosensors and photodetectors because of excellent conductivity, transparency, and nanofabrication adaptability [33,34,35]. While this work aims to demonstrate the basic concept of cell surface charge mapping using photoelectric effects, submicron or nanoscale microelectrodes based on graphene or molybdenum disulfide can be fabricated to achieve excellent charge transfer with higher resolution. The spatial resolution and the sensitivity of surface charge mapping can thus be improved.

## 4. Conclusions

We demonstrated a microelectrode array deposited on an ITO layer for mapping of a single cell’s surface charge. When a cell is placed on the microelectrodes, its local potential/surface charge leads to an increased charge transfer to the microelectrode-ITO layer and higher photon absorption and thus causes a reduction in the reflected light intensity. By measuring the intensity change of the light reflected from the microelectrode array, the surface charge distribution of the cell can be determined. This surface charge mapping method does not need physical or chemical modification of the cells. We first validated the feasibility of the surface charging mapping method using 30 μm microparticles. In addition, we performed surface potential mapping on two types of cells (HDFs and hMSC). From the mapping, the average surface potentials of these two types of cells were obtained, which were in good agreement with the electrophoretic light scattering measurements. With its simple structure, this microelectrode array can be used for surface charge mapping and monitoring of a variety of cells, biomaterials, and tissue organisms.

## Data Availability

Available upon reasonable request.
